# ROHHAD syndrome without rapid-onset obesity: A diagnosis challenge

**DOI:** 10.3389/fped.2022.910099

**Published:** 2022-08-31

**Authors:** Blandine Desse, Antoine Tran, Mathilde Butori, Sarah Marchal, Michael Afanetti, Sébastien Barthélemy, Etienne Bérard, Elisabeth Baechler, Stéphane Debelleix, Marie-Emilie Lampin, Julie Macey, Bruno Massenavette, Julie Harvengt, Ha Trang, Lisa Giovannini-Chami

**Affiliations:** ^1^Pediatric and Neonatology Department, Hopital de Grasse, Grasse, France; ^2^Pediatric Emergency Department, Hôpitaux pédiatriques de Nice CHU-Lenval, Nice, France; ^3^Pediatric Gastroenterology Department, Hôpitaux pédiatriques de Nice CHU-Lenval, Nice, France; ^4^Pediatric Pulmonology and Allergology Department, Hôpitaux pédiatriques de Nice CHU-Lenval, Nice, France; ^5^Pediatric Intensive Care Unit, Hôpitaux pédiatriques de Nice CHU-Lenval, Nice, France; ^6^Pediatric Nephrology Department, Hôpitaux pédiatriques de Nice CHU-Lenval, Nice, France; ^7^Pediatric Endocrinology Department, Hôpitaux pédiatriques de Nice CHU-Lenval, Nice, France; ^8^Pediatric Pulmonology Department and Cystic Fibrosis Center, CHU de Bordeaux, Bordeaux, France; ^9^Pediatric Intensive Care Unit, CHU de Lille, Lille, France; ^10^Respiratory Medicine and Cystic Fibrosis Center, CHU de Bordeaux, Bordeaux, France; ^11^Intensive Care Unit, Hospices Civils de Lyon, Hopital Femme Mere Enfant, Bron, France; ^12^Department of Human Genetics, Sart-Tilman, Liège, Belgium; ^13^Pediatric Sleep Center, Hopital Universitaire Robert Debre, Paris, France; ^14^Université Côte d'Azur, Nice, France

**Keywords:** ROHHAD syndrome, hypothalamic syndrome, child, central hypoventilation, dysautonomia

## Abstract

**Background:**

ROHHAD syndrome (Rapid-onset Obesity with Hypothalamic dysfunction, Hypoventilation and Autonomic Dysregulation) is rare. Rapid-onset morbid obesity is usually the first recognizable sign of this syndrome, however a subset of patients develop ROHHAD syndrome without obesity. The prevalence of this entity is currently unknown. Alteration of respiratory control as well as dysautonomic disorders often have a fatal outcome, thus early recognition of this syndrome is essential.

**Material and methods:**

A retrospective, observational, multicenter study including all cases of ROHHAD without rapid-onset obesity diagnosed in France from 2000 to 2020.

**Results:**

Four patients were identified. Median age at diagnosis was 8 years 10 months. Median body mass index was 17.4 kg/m^2^. Signs of autonomic dysfunction presented first, followed by hypothalamic disorders. All four patients had sleep apnea syndrome. Hypoventilation led to the diagnosis. Three of the four children received ventilatory support, all four received hormone replacement therapy, and two received psychotropic treatment. One child in our cohort died at 2 years 10 months old. For the three surviving patients, median duration of follow-up was 7.4 years.

**Conclusion:**

ROHHAD syndrome without rapid-onset obesity is a particular entity, appearing later than ROHHAD with obesity. This entity should be considered in the presence of dysautonomia disorders without brain damage. Likewise, the occurrence of a hypothalamic syndrome with no identified etiology requires a sleep study to search for apnea and hypoventilation. The identification of ROHHAD syndrome without rapid-onset obesity is a clinical challenge, with major implications for patient prognosis.

## Introduction

ROHHAD syndrome (Rapid-onset Obesity with Hypothalamic dysfunction, Hypoventilation and Autonomic Dysregulation) is a rare disease that was initially described in 1965 under the name “Late-Onset Central Hypoventilation Syndrome with Hypothalamic Dysfunction (LO-CHS/HD)” (1). Subsequently, just over 150 cases have been reported in the literature (2). Recognition of this sydrome constitutes a challenge for clinicians. In 2007, the Ize-Ludlow team (3) suggested diagnostic criteria for ROHHAD combining: (i) rapidly evolving morbid obesity in the first years of life, (ii) signs of hypothalamic dysfunction, (iii) central hypoventilation occurring in childhood, (iv) signs of dysautonomia.

Due to the association with neural crest tumors in 15–50% of cases, the hypothesis of a paraneoplastic etiology has been suggested, but not confirmed (2, 4). The ROHHANET syndrome acronym (ROHHAD and NEural Tumors) have been suggested to describe the entity but ROHHAD remains the preferred term because it accounts for those with and without tumors. No candidate gene has been identified *via* exome sequencing (3–6) and the hypothesis of a monogenic etiology has been challenged by the description of a case in only one of a pair of monozygotic twins (7). The dysimmunity hypothesis seems probable, in particular with the presence of oligoclonal bands in the cerebrospinal fluid of two patients (8) and of antihypothalamic and antipituitary autoantibodies in the serum and cerebrospinal fluid of another patient (9).

Morbid obesity with rapid onset driven by hypothalamic dysfunction is the first recognizable sign of this syndromic sequence in 80–100% of ROHHAD cases. This occurs early in life (median 3 years old) and has been described by Ize-Ludlow as one of the mainstays of the diagnosis (3, 10). A better prognosis of pathology in ROHHAD is associated with an early diagnosis (11).

The main objective of this work was to describe the characteristics of ROHHAD syndrome without rapid-onset morbid obesity (RO) at diagnosis, in cases retrieved from the French cohort of Disorders of Ventilatory Control, registered in the RespiRare® (French Reference Center for Rare Lung Diseases) database. The secondary objective was to compare this cohort with a recent systematic review of cases of ROHHAD with RO (10).

## Methods

### Population

All cases of ROHHAD without RO who were <18 years old at the time of diagnosis, and registered in the “RespiRare®” French national pediatric database for rare lung diseases, were included through a multicenter national, retrospective study. The RespiRare® database was established in 2006, and includes 33 French “competence centers.” Each patient and/or their legal representative gave informed consent prior to their details being entered in the database. For cases that were not listed in the database, data were retrieved by contacting all the French competence centers for rare lung diseases asking for cases followed between 2000 and 2020. The database was approved by the French National Data Protection Authorities and the study was approved by the Institutional Review Board of the “Société de Pneumologie de Langue Française” (France).

The inclusion criteria were:

- hypoventilation confirmed on a polysomnography recording, without pulmonary or cardiac etiology, nor brainstem lesion
- at least one of the following signs of hypothalamic dysfunction: growth hormone deficiency, diabetes insipidus, puberty advance or delay, hypogonadism, hyperprolactinemia, central hypothyroidism, corticotropic insufficiency
- at least one symptom of dysautonomia (hypo/hyperthermia, cold hands and feets, sweating dysregulation, bradycardia, syncopes, orthostatic hypotension, bladder dysmotility, digestive dysmotility (alternating between constipation, diarrhea), strabismus, ptosis, altered perception of pain, …)
- absence of rapid-onset obesity defined by a body mass index greater than or equal to IOTF-30 when diagnosing ROHHAD. IOTF-30 (International Obesity Task Force) has been defined as child centile curve of BMI corresponding to an adult BMI cut-off points of 30 kg/m2 (12).

### Data collection

Data regarding clinical features at the time of diagnosis, investigations, treatment, and outcome were collected using a standardized questionnaire sent to each coordinator physician at the 33 French centers that had declared a patient.

The following patient data, as at diagnosis, were collected and analyzed further: *sociodemographic* (age, sex, weight, height, body mass index, siblings, personal and family history); *diagnostic* (clinical signs, date of onset of specific signs); *hypothalamic involvement* (type of involvement, laboratory parameters, pituitary MRI results); *hypoventilation* (CO_2_ response test, results of polysomnography at diagnosis, results of functional respiratory examinations, results of the walking test or stress test, results of the blood gas); *autonomic nervous system damage* (results of ECG and blood pressure holters, cardiac ultrasound data); *behavioral disorders*, if present; *neurological damage*, if present (cerebrospinal fluid analysis, brain MRI); *neuroendocrine tumor*, if present (date of diagnosis, nature and location of the tumor, type of treatment and effect on symptoms, data from thoracic and abdominal imaging); *therapeutic measures* (respiratory treatment: type of ventilatory support, duration, need for a tracheostomy; immunomodulatory treatment: administration of intravenous immunoglobulins, immunosuppressive treatment, type of molecule used, dose and start date; hormonal treatment: control of water intake, molecules used; surgical treatment: adenoidectomy, tonsillectomy, tumor excisional surgery, others; psychiatric treatment); *outcome* [reasons and date of hospitalization (excluding follow-up)]; *education and social support*; *date of the latest evaluation*; *date and cause of death*, if applicable.

### ROHHAD with RO cohort

Data from the systematic review of cases of ROHHAD with RO published in the literature (13) were extracted. Additional data not presented in the publication were kindly provided to us by the first author.

### Statistics

Descriptive analysis of the sample and figures were performed with R software (www.r-project.org). Data are expressed as median and interquartile range for quantitative variables. The comparison of the quantitative variables was carried out with the Wilcoxon-Mann-Whitney test. The results were considered statistically significant when the *p*-value was < 0.05.

## Results

Ten cases were identified from the initial screening of the French database. Of these ten cases, five were excluded because of obesity at the time of diagnosis, after checking BMI on IOTF-30 curves. Of the five remaining eligible cases, one was excluded due to a clinical and paraclinical presentation closer to encephalitis.

### Demographic data

Our study population thus included four children, all male. Consanguinity was not present in two of patients, with data not available for the other two.

Three of the four children had a significant family history. Two parents had dysthyroidism and one parent had been treated for Hodgkin's disease at the age of 9 years. The sibling of one patient was followed by ENT for recurrent infections, while the sibling of another patient presented with Prader-Willi syndrome (PWS).

### Diagnosis

**Figure 1:**
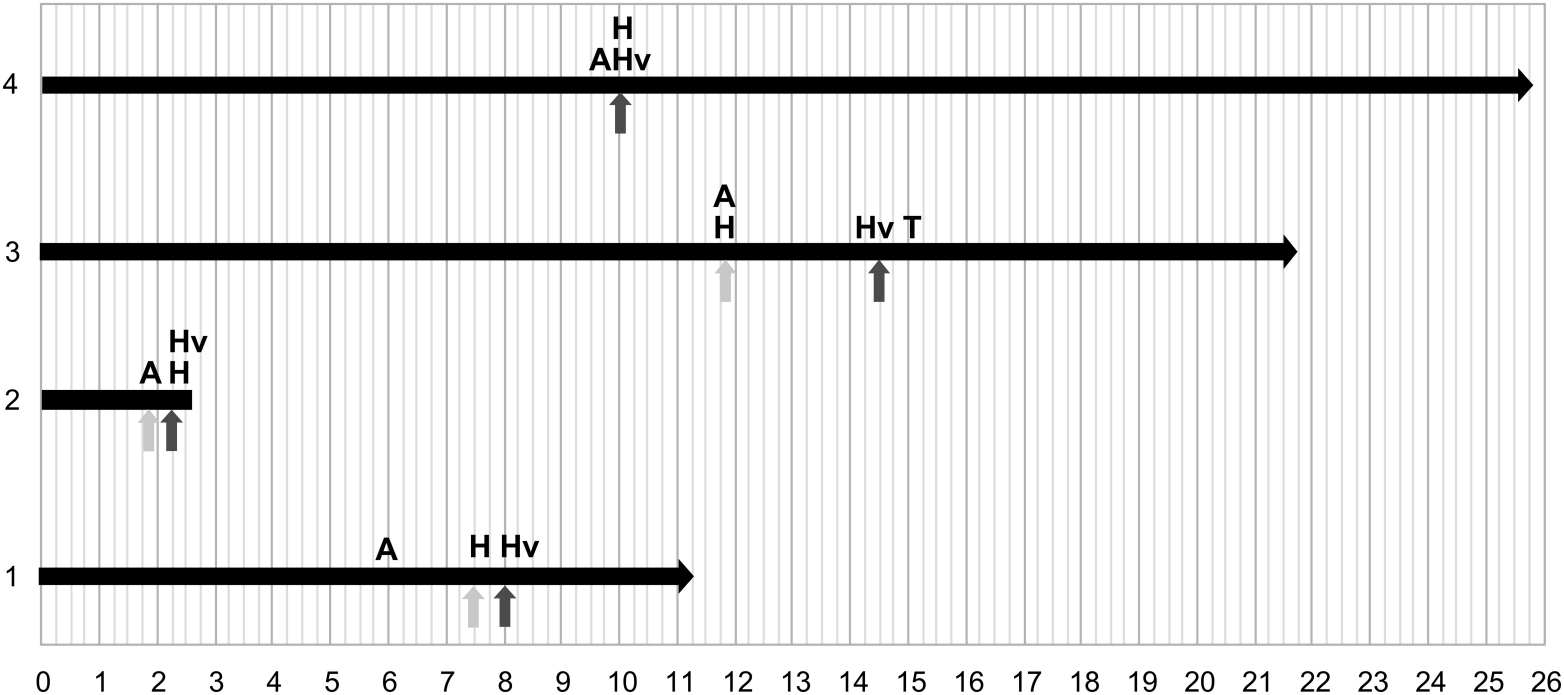
Chronology of onset of clinical signs. 1–4 correspond to each patient of the cohort and axial axis to the age at onset of symptoms in years. The end of the black horizontal arrow corresponds to the last follow-up of the patient, or patient death. H represents hypothalamic involvement, A dysautonomia, Hv hypoventilation, T the occurrence of a tumor. The light-gray arrows indicate the time of the first hospital referral and the dark-gray arrows the time of diagnosis.

**Figure 2:**
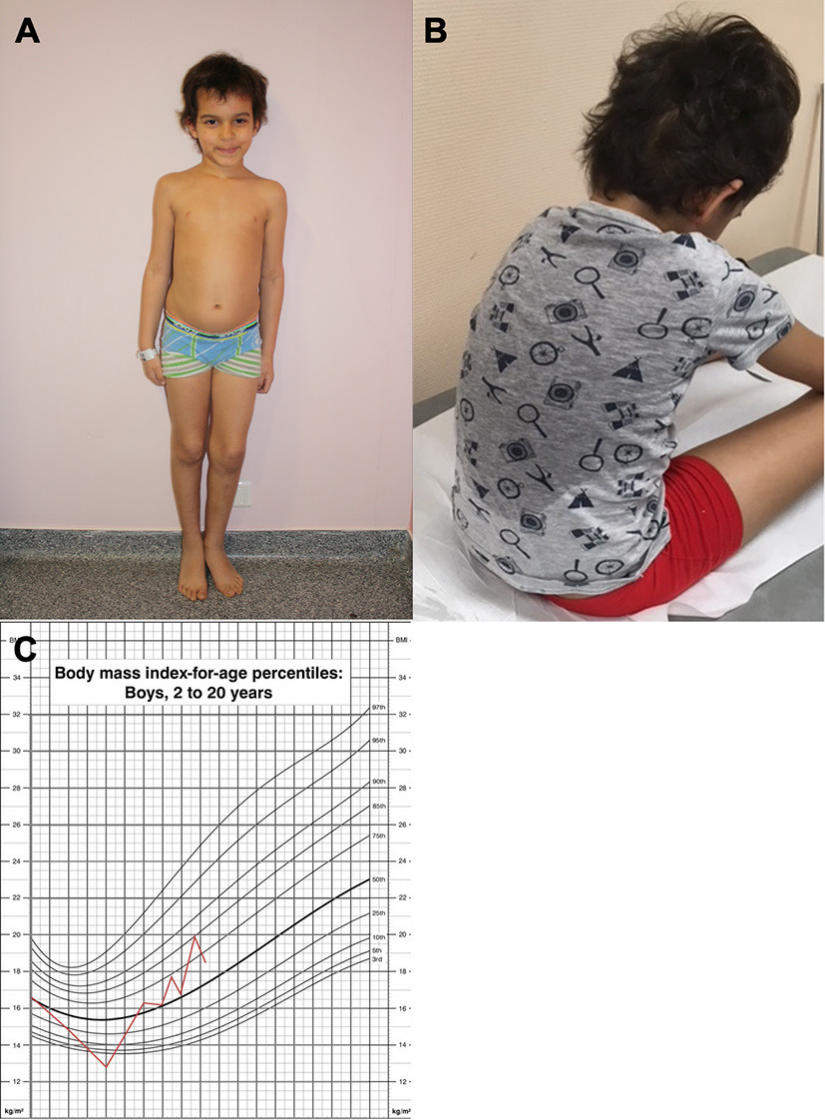
Pictures of a patient of the cohort. Panel showing **(A)** normal BMI at diagnosis **(B)** excessive sweating **(C)** follow-up of BMI.

The median age at diagnosis of ROHHAD syndrome was 8 years 10 months [2 years 9 months−12 years 7 months]. The first signs of the disease, defined retrospectively, were different depending on the child: hypersomnia; association of fever and excessive sweating; panhypopituitarism; seizure in the context of hypothermia. Hospitalization following onset of the first symptoms occurred for three of four patients, median 3 months [3–3.1] prior to diagnosis. These three children were, respectively, hospitalized for hypernatremic coma, hypoxemic pneumonia, and prolonged unexplained fever with constant strabismus. These hospitalizations did not lead to the diagnosis of ROHHAD. The chronology of onset of clinical signs is summarized in Figure 1. The median body mass index was 17.4 kg/m^2^ [16.5–19.35] (Figures 2A–C).

The median time to diagnosis of ROHHAD syndrome was 4.4 months [2.8 months−12.6 months]. The maximum time to diagnosis was 35.4 months. Two diagnoses were evoked by a pediatric pneumopediatrician, the other two, respectively, by a pediatric intensivist and a pediatric endocrinologist. All four patients were screened for any PHOX2B pathogenic variant involved in central hypoventilation syndrome (14). No patient had a pathogenic variant in this gene. An exome sequencing was performed in one patient of the cohort and no pathogenic variant was identified.

### Hypothalamic dysfunction

**Figure 3:**
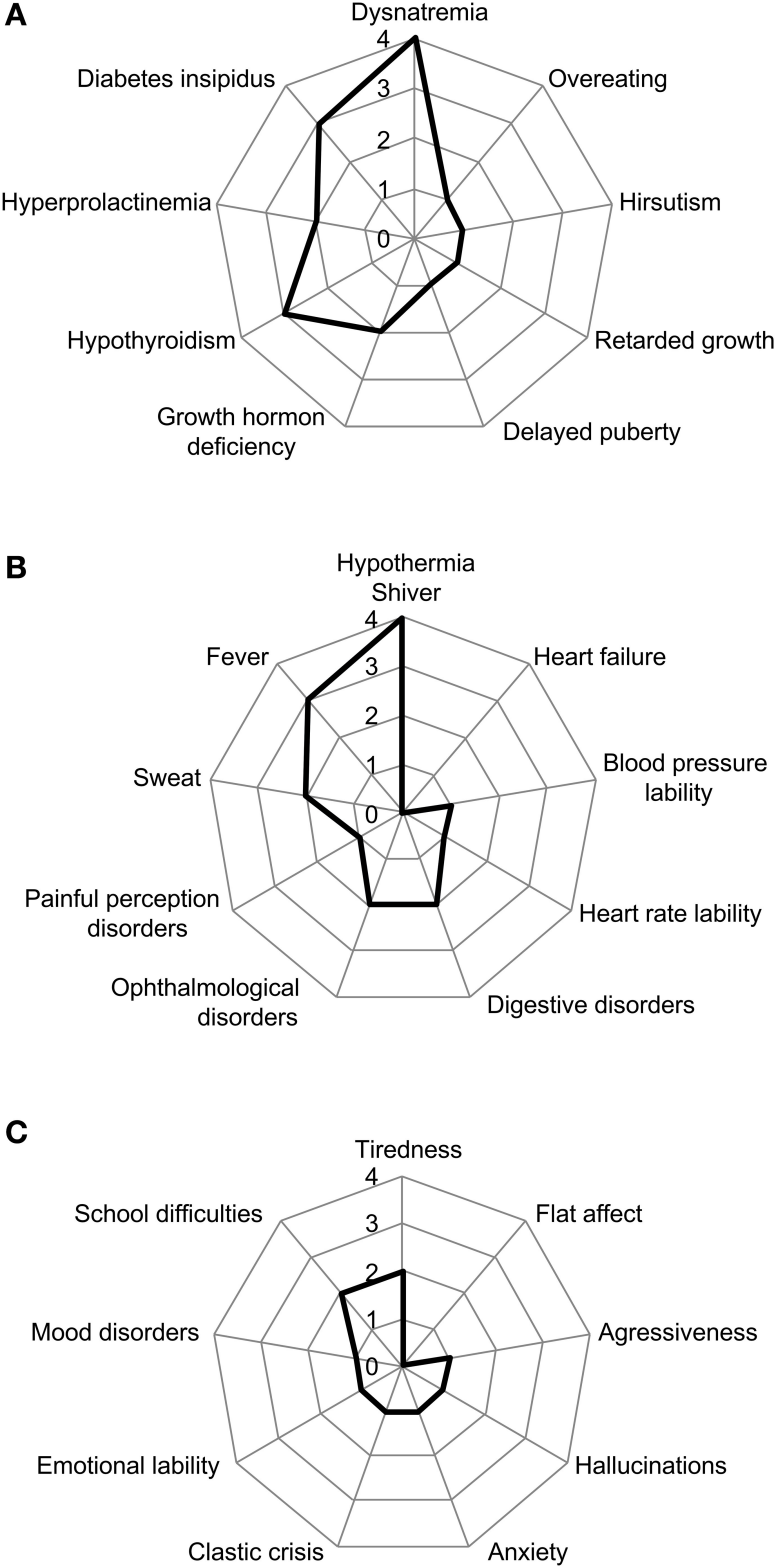
Distribution of main clinical signs. Radar plots showing mains clinical signs encountered concerning the fields of **(A)** hypothalamic dysfunction, **(B)** dysautonomia, **(C)** behavioral disorders.

All four children showed hypothalamic dysfunction, with a median age at onset of 8 years 8 months [6 years 2 months−10 years 3 months]. These signs appeared a median of 2 months before the diagnosis of ROHHAD [1–12 months]. The median number of manifestations of hypothalamic dysfunction per child was 5 [3.75–6.5] (Figure 3A).

### Hypoventilation

All four children showed hypoventilation, with a median age at onset of 8 years 9 months [6.4–11]. Hypoventilation led to the ROHHAD diagnosis. One child had no respiratory complaints. Two of the four patients presented with sleep apnea. Two of the four children presented episodes of respiratory distress in an infectious context. None of the four patients presented with an attack of cyanosis. All children underwent polysomnography. For 2 patients, the clinical signs of hypoventilation prompted polysomnography. For 2 patients, hypothalamic dysfunction was the main prompter of polysomnography. This was performed at a mean age of 9.3 years [6.5–11.9], 4.5 months after first respiratory symptoms leading to diagnosis [0.4–9]. The results of the initial polysomnography are summarized in Table 1. This initial polysomnography showed hypoventilation in the four cases. Isolated OSA before of onset hypoventilation was not diagnosed in our cohort. Only one patient underwent a test of ventilatory response to carbon dioxide, which was abnormal. Respiratory functional explorations were performed in three children in our cohort, showing a restrictive syndrome for one and being normal for the two others. Only two children in our cohort underwent a stress test, one was normal and the other sub-maximal, nevertheless showing limited aerobic capacity for exercise. Venous blood gas results were available for three of the four patients showing a median capnia of 60 mmHg [56.5–85], median pH of 7.30 [7.27–7.34], median alkaline reserve of 40 mmol/L [33.7–43]. Nasofibroscopy could be performed in two of our patients and was normal.

**Table 1:** Results of the initial polysomnography.

	**Patient 1**	**Patient 2**	**Patient 3**	**Patient 4**
**Recording**	Night	Nap	Night with ventilatory support	Night
**Age (years)**	7.8	2.4	15.2	10.8
**Paradoxical sleep (%)**	20	na	22.7	na
**Apnea-hypopnea index (/hour)**	8	3.5	2.1	4
**Average SpO**_**2**_ **(%)**	94.2	90.8	na	94
**Minimal SpO**_**2**_ **(%)**	na	68	83	90
**Average TcPCO**_**2**_ **(mmHg)**	48	na	na	50
**Maximal TcPCO_2_** **(mmHg)**	55	60	50	52
**% time with TcPCO_2_>** **50 mmHg**	38	na	0	10

### Dysautonomia

All four children showed dysautonomia, with a median age at onset of the first signs at 7 years 10 months [4.8–10.3]. These signs appeared 15 months before diagnosis of ROHHAD [3.8–27.6]. The median number of clinical signs indicating dysautonomia was 3.5 per patient [1.75–5.75] (Figure 3B). No patient presented with cardiac rhythm disorders and no patient presented pulmonary hypertension.

### Behavioral disorders

All four children showed behavioral disorders, with a median age at onset of 6 years [4.2–10.1]; 2.9 months [1.3–13.9] before ROHHAD diagnosis (Figure 3C).

### Neurological involvement

Three out of the four children presented with neurological involvement, at a median age of 9 years 10 months; 0.26 months [0.13–1.7] after ROHHAD diagnosis. Two of these three patients presented with initial disturbances of consciousness. One patient presented with febrile convulsions at the time of diagnosis. Two children presented with trouble sleeping and one patient presented with attacks of ataxia with catatonia. The cerebrospinal fluid of two children was analyzed, showing no hyperproteinorachia, but seven extra oligoclonal bands in one child. Of two children who underwent electroencephalographic recordings, one was normal, while one patient showed slow bilateral activity. Brain MRI results, performed on average 1.7 months after diagnosis, were available for three patients and did not show any abnormalities, notably of hypothalamic-pituitary structures.

### Tumor involvement

Only one child developed a tumoral lesion, in the psoas area, during the course of his disease. This lesion has never been biopsied and is monitored regularly without any sign of progression to date.

### Therapeutic care

Three of the four children received ventilatory support, which was noninvasive and nocturnal-only in two cases, and invasive *via* tracheostomy for one. A single patient received intravenous corticosteroid pulses, intravenous immunoglobulins at a dose of 2 g/kg four times, rituximab at a dose of 375 mg/m^2^/week five times, three sessions of plasmapheresis, ten sessions of immunoadsorption, and four injections of cyclophosphamide. This was the patient with a severe form of ROHHAD with a rapidly unfavorable course, and for whom analysis of the cerebrospinal fluid had identified the oligoclonal bands. Two of the four patients received psychotropic treatment (risperidone, clonidine, lorazepam). The fluid intake of three of the four patients was monitored. All patients received opotherapy (L-thyroxine 3/4, desmopressin $\frac{3}{4}$, Growth hormone 2/4, hydrocortisone hemisuccinate and testosterone 1/4). The median number of molecules was 2 [2–2.5].

### Follow-up

Two children were readmitted to hospital for decompensation subsequent to the hospitalization leading to the ROHHAD diagnosis; one patient for accidental drug intoxication with risperdone, while the other case was rehospitalized four times because of episodes of paralysis of the lower limbs, either associated, or not, with hypothermia.

One child in our cohort died at age 2 years 10 months, 5 months after being diagnosed with ROHHAD. The death occurred as a result of limited care in a context of respiratory decompensation. For the three surviving patients, the median duration of follow-up was 7.4 years [5.3–11.6]. The schooling was arranged for two children.

None of these three patients developed obesity during this follow-up period.

### Comparison with data from the literature of ROHHAD with RO

We compared the age of onset of the different symptom groups constituting ROHHAD syndrome with data from the systematic review of the literature (10). These data are represented on the Kaplan-Meier curves in Supplementary Figure S1. The disorders seem to occur later in our population, in particular for hypothalamic disorders. However, this difference was not statistically significant due to our limited number of patients. We moreover compared the frequency of each symptom in the two cohorts (Supplementary Table 1). Hypernatremia and diabetes insipidus were significantly more frequent in our cohort and tumor of neural crest significantly lest frequent. Unusual symptoms were described in our cohort such as ataxia, paralysis of the lower limbs and a begnin tumor of the psoas. Our cohort was moreover exclusively constituted of boys.

## Discussion

In order to make the diagnosis easier, in 2007 (3) the acronym ROHHAD was proposed to describe the clinical criteria for syndromic diagnosis based on the chronology of symptom onset. Rapidly progressive obesity was described as being the first warning sign of this pathology. We now describe the first cohort of children who were not obese when diagnosed with ROHHAD and who did not develop obesity during follow-up.

In a systematic review of recent literature focusing on the chronology of symptom onset (10), the median age of onset of this obesity was 3.1 years (2.8–4), sometimes preceded by a phase of hyperphagia appearing at the mean age of 3 years (2–3.6). This review (which retains obesity as necessary for the diagnosis) collected 43 cases from the literature for which chronological data were available. The review reported that rapid-onset obesity was always the first sign in this cohort. No study in the literature has, to date, specifically described the entity of ROHHAD without obesity at diagnosis. Its prevalence is therefore very difficult to assess.

Of the fifteen children in the Ize-Ludlow study population (3), obesity was not the inaugural symptom in two children (13.3%). However, both of these children progressed to major obesity within 1 to 5 years. The alveolar hypoventilation in these two cases followed obesity, and one could imagine that the diagnosis was not made on the initial symptoms of dysautonomia, the procession of other symptoms having arisen around obesity.

Likewise, in the cohort of De Pontual et al. (15), rapid-onset obesity was not the first sign of the pathological sequence in three of the thirteen patients included (23%). Only one patient in the cohort did not progress to obesity. However, looking closely at the data presented in this paper, this 2-year-old child was already obese at diagnosis (BMI 20.5), even if not as massively obese as the other patients.

Descriptions in the literature often describe onset of obesity preceded by a period of overeating. In our sample, periods of transient overeating were described in one patient, without significant weight gain. A recent and rapid weight gain was described for the youngest patient in our cohort, but without mention of obesity. The outcome for this patient was unfavorable over a few months and it is likely that weight gain was limited by the clinical condition. In our cohort, the median age at ROHHAD diagnosis was 8 years 10 months, later than the median age described in the literature (4.75 years) (9). The prolonged follow-up of our surviving patients (median of 7.4 years) suggests that they have a specific form of ROHHAD syndrome that does not progress to obesity.

The average time to ROHHAD diagnosis in our population was 4.4 months. The lack of obesity led to diagnostic uncertainty. Several articles in the literature indicate consideration of ROHHAD syndrome when obesity occurs, especially when it is isolated, early, or severe (16, 17), making this manifestation an essential sign for a ROHHAD diagnosis. However, even when early-onset obesity occurs, there can be a significant delay from presentation to diagnosis of ROHHAD syndrome, notably in countries with high prevalence of morbid obesity like the United States. A diagnostic delay of more than 10 years in a young 19-year-old woman was recently reported. This patient had been suffering from severe morbid obesity since the age of 3 years, she presented obstructive sleep apnea at 3 years old, hypoventilation at 5 years old and hypothalamic deficit at 7 years old. The diagnosis of ROHHAD was made at the age of 19 years when severe dysautonomic signs appeared after three visits to the intensive care unit (18). The story is similar for the patient described by Jalal Eldin et al. (19) in 2019. Apart from the absence of obesity, patients in our study population met the diagnostic criteria for ROHHAD syndrome.

The earliest signs found retrospectively in our population were signs of autonomic dysfunction. These were described around a mean age of 6 years and were mostly disorders of thermal regulation. These data are consistent with data from the 2020 literature review (13). No patients of our cohort presented with tumor of the neural crest.Dysautonomia is rare in pediatrics and is often difficult to identify due to the large polymorphism and non-specific nature of clinical signs. Brain damage is the main underlying cause of dysfunction (20). In the three of our patients who underwent brain imaging, findings were normal and it is likely that an infectious etiology was initially suggested to explain the thermal regulation disorders. ROHHAD syndrome should be mentioned when signs of dysautonomia appear in the absence of a history suggesting brain damage or in the event of normal brain imaging.

In our population, on average 21 months after the signs of dysautonomia, the signs of hypothalamic dysfunction appeared, at a median age of 8 years 8 months. In a recent review of the literature, signs of hypothalamic dysfunction were present in 41 of 43 patients with a median age of onset of 4 years (10). Hyperprolactinemia and dysnatremia were the earliest signs. Hypernatremia and diabetes insipidus were significantly more frequent in our cohort compared to Harvengt series. Central hypothyroidism is present in 30% of ROHHAD patients and growth hormone deficiency in approximately half of patients (21, 22). In a study published by Bougnères et al. (17) in 2008, describing endocrine abnormalities in patients diagnosed with ROHHAD, all of the patients presented blood serum abnormalities, hyperprolactinemia and dysthyroidism. The endocrine profile of our patients is similar, apart from early and rapidly developing obesity, but dyshypothalamic disorders appear to occur later.

Psychiatric and behavorial problems were very similar to those encountered in ROHHAD with RO cohorts. Neurological symptoms such as ataxia and paralysis of the lower limbs in a context of dysautonomia and mild rhabdomyolysis have never been described to our knowledge in previously published case reports.

Hypoventilation is the cardinal symptom of the non-obese ROHHAD entity. In fact, the clinical signs of hypoventilation or the results of polysomnography made it possible to make the diagnosis in all of our patients faced with a combination of hypoventilation - dysautonomia - hypothalamic disorders. In our population, hypoventilation occurred at a median age of 8.7 years [6.4–11]. A retrospective study published in 2016 (4) showed that ROHHAD patients initially presented with obstructive sleep apnea and that hypoventilation only appeared secondarily, associated with respiratory irregularity during wakefulness. In this study, the median age of onset of hypoventilation was 7.2 years. In the study by Harvengt et al. (13), there was also a lag between the age of observation of obstructive sleep apnea (4 years) and the age at diagnosis of hypoventilation (5.3 years). Obstructive sleep apnea is not a diagnostic criterion for ROHHAD, but it seems to occur before hypoventilation and could indicate this diagnosis more quickly. Isolated OSA before of onset hypoventilation was not diagnosed on the initial polysomnography in our cohort in contrast to prior studies in ROHHAD. The respiratory clinical signs were mainly a symptomatology of sleep apnea or acute decompensations in an infectious context. However, diagnosis was only made by a pediatric pulmonologist in two of the four cases. The diagnosis can be suggested by intensivists during acute episodes of decompensation (23) or during severe dysautonomia, by pediatric pulmonologists during diagnostic exploration for hypoventilation or by pediatric endocrinologists in the event of hypothalamic involvement. It is clear that these signs must be interpreted together in order to reach the diagnosis, provided that this syndromic association is known by these different specialists.

To date, no curative treatment for ROHHAD exists. As for diagnosis, the management of these children must be multidisciplinary. Ventilatory impairment and dysautonomia impact negatively on prognosis for these children. The installation of adapted ventilatory support makes it possible to limit morbidity and to prevent the occurrence of cardiorespiratory arrests. The association with neural crest tumors in more than half of the life-threatening cases (13) indicates oncological screening in these children early in the treatment and on a regular basis. Efficacy of the immunomodulatory treatments varies according to the studies (24–27). The correction of endocrine disorders and monitoring of water and sodium intake if necessary, as well as dietary management, are essential. Patients with ROHHAD syndrome may also present behavioral disorders of multifactorial origin (28, 29), for which management and care should not be forgotten. Psychological and social support for the family must be organized. As ROHHAD syndrome is little known, emergency departments must be informed in advance of the risk of cardiopulmonary arrest in these children and of the actions to be taken.

The prognosis for this pathology can be severe. One of our four patients died. In the literature, the mortality rate varies from 6% (3) to 30% (17) depending on the study. The prognosis and quality of life of surviving children has not been established. There are a few articles reporting ROHHAD cases that have become adults. One patient presented with severe metabolic syndrome and hepatocellular carcinoma at the age of 26 years, with fatty liver disease (19). A 22-year-old patient presented with acute respiratory failure on non-invasive ventilation (30).

Our study is the first to describe the clinical and evolutionary characteristics of patients with genuine ROHHAD syndromes without RO (Rapid-onset Obesity). Persistent absence of obesity during follow-up constitutes a newly described phenotype of the disease, even if endotype remains unknown. An understanding of the pathogenesis of this entity would be noteworthy in order to classify this entity as a sub-type of ROHHAD or as a really new entity. Knowledge of this particular entity will allow early recognition of affected patients and avoid diagnostic errancy that could be life-threatening. Management of the various conditions and early detection of associated tumors will limit the associated morbidity and increase the duration and quality of life of these patients.

The main limitation of our study is the small size of our population, probably linked to the lack of knowledge of this entity, non-consensual diagnostic criteria, retrospective design of the study and therefore the non-recognition of affected patients. Moreover, obstructive and central apnea-hypopnea index were not available as some centers reported only a global hypopnea index and polysomnography reports did not determine if hypoventilation was worse in non-REM sleep or REM sleep. Larger studies are needed to more accurately describe the typical profile of patients with ROHHAD without RO. These studies may also make it possible to progress with knowledge of the pathophysiology of this syndrome.

## Conclusion

ROHHAD syndrome without RO (Rapid-onset Obesity) appears to be a separate, later onset entity. Its identification is difficult due to the absence of the most easily identifiable and earliest sign in the sequence of classic ROHHAD syndrome. It is likely that without being described in the literature, this syndrome is underdiagnosed. Given its severity, it seems important to discuss this diagnosis in the context of cases where signs of dysautonomia appear in the absence of a history suggesting brain injury or the appearance of several signs of pituitary dysfunction. In the absence of a diagnostic or genetic test, performing repeated polysomnography in these situations could better identify these patients and therefore improve their prognosis. International studies are needed to better describe the clinical and progressive features of this syndrome.

## Data availability statement

The original contributions presented in the study are included in the article/Supplementary material, further inquiries can be directed to the corresponding author.

## Ethics statement

The studies involving human participants were reviewed and approved by société de Pneumologie de Langue Française IRB. Written informed consent to participate in this study was provided by the participants' legal guardian/next of kin.

## Author contributions

LG-C and HT designed the study, critcally revised the first draft, and approved the final draft. BD collected the data and wrote the first draft and approved final draft. AT, JH, MB, EBe, EBa, SM, MA, SB, SD, M-EL, JM, BM, HT, and LG-C provided data, critically revised the initial draft, and approved the final version.

## Funding

Hôpitaux pédiatriques de Nice CHU-Lenval provide funds for open access publication fees.

## Conflict of interest

The authors declare that the research was conducted in the absence of any commercial or financial relationships that could be construed as a potential conflict of interest.

## Publisher's note

All claims expressed in this article are solely those of the authors and do not necessarily represent those of their affiliated organizations, or those of the publisher, the editors and the reviewers. Any product that may be evaluated in this article, or claim that may be made by its manufacturer, is not guaranteed or endorsed by the publisher.

## Supplementary material

Supplementary Figure S1 Kaplan Meier curves of clinical signs age of onset concerning **(A)** hypoventilation, **(B)** hypothalamic dysfunction, **(C)** autosomic dysregulation. In black, data from our population of ROHHAD without RO; in dotted lines, data from the study by Harvengt et al. (13) of ROHHAD with RO.
