# Supplementary figures and images for: ROHHAD syndrome without rapid-onset obesity: A diagnosis challenge
Source: Front Pediatr. 2022 Aug 31;10:910099. doi: 10.3389/fped.2022.910099 (PMC9471950; doi:10.3389/fped.2022.910099)

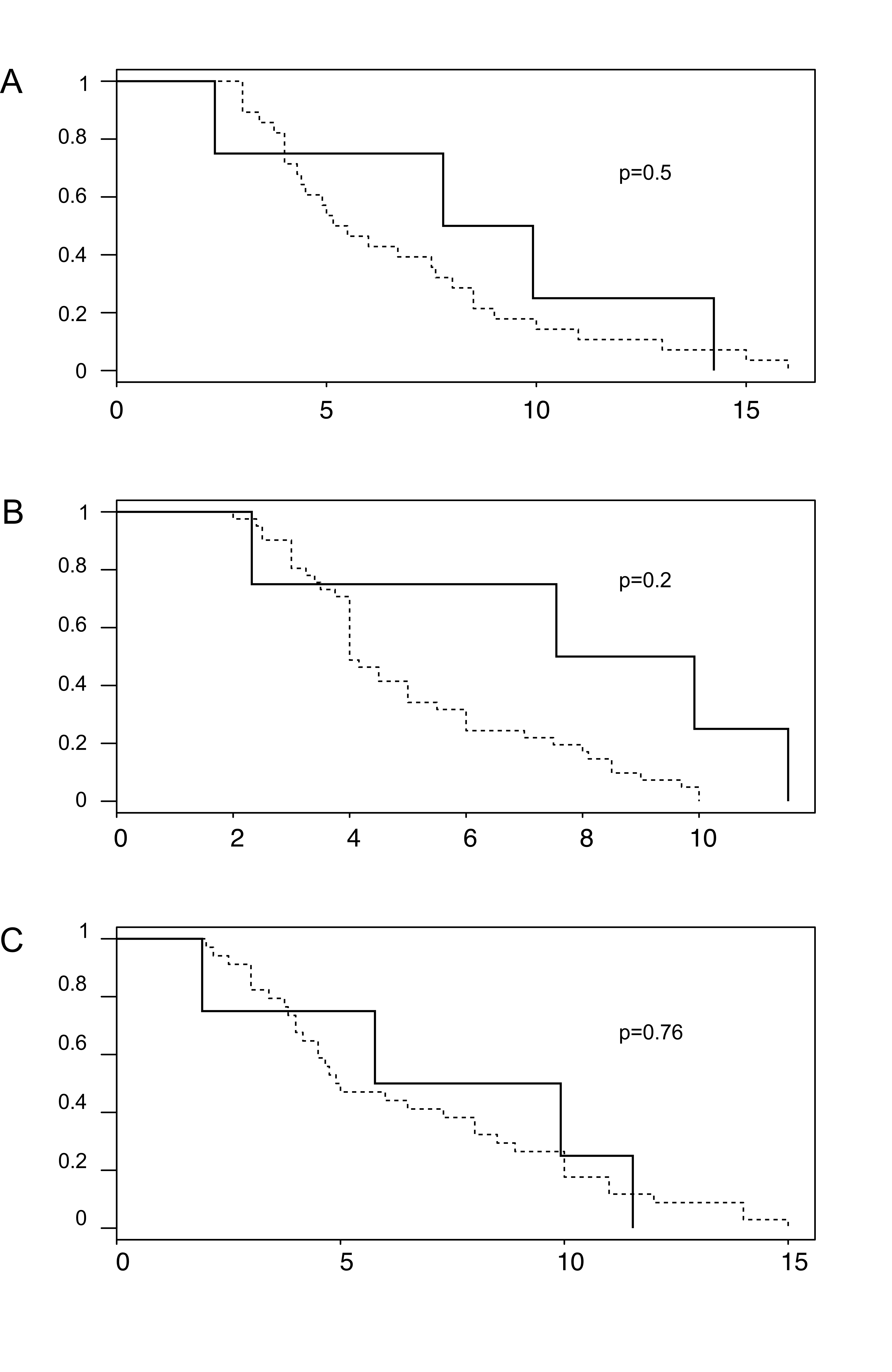

Supplement: Supplementary Figure S1 — Kaplan Meier curves of clinical signs age of onset concerning (A) hypoventilation, (B) hypothalamic dysfunction, (C) autosomic dysregulation. In black, data from our population of ROHHAD without RO; in dotted lines, data from the study by Harvengt et al. (13) of ROHHAD with RO. [file Image_1.TIF]
