# Supplementary material for: ROHHAD syndrome without rapid-onset obesity: A diagnosis challenge
Source: Front Pediatr. 2022 Aug 31;10:910099. doi: 10.3389/fped.2022.910099 (PMC9471950; doi:10.3389/fped.2022.910099)
Supplement: Supplementary file 2 [file Table_1.docx]

**Supplemental Table 1**

|  | **Harventgt cohort [11]** | **Our cohort** | p-value |
| --- | --- | --- | --- |
| number of patients | 43 | 4 |  |
| sex ratio | 8/29 | 4/0 | **0.005** |
| **HYPOTHALAMIC** |  |  |  |
| hyperphagia | 23 | 1 | 0.348 |
| RO | 43 | 0 | **<0.001** |
| hyperPRL | 22 | 2 | 1 |
| hypothyroidism | 12 | 3 | 0.089 |
| hypernatremia | 19 | 4 | **0.049** |
| diabetes insipidus | 9 | 3 | **0.046** |
| hyponatremia | 4 | 0 | 1 |
| GH deficiency | 12 | 2 | 0.512 |
| precocious puberty | 5 | 0 | 1 |
| delayed puberty | 4 | 1 | 0.372 |
| **DYSAUTONOMIA** |  |  |  |
| thermal dysregulation | 23 | 4 | 0.126 |
| cold ands and feet | 6 | nd |  |
| sweating alteration | 10 | 2 | 0.266 |
| bradycardia | 8 | 1 | 1 |
| syncopes | 1 | 0 | 1 |
| PCMK implatation | 3 | 0 | 1 |
| altered perception of pain | 7 | 1 | 0.539 |
| ophtalmological disorders | 7 | 2 | 0.160 |
| digestive dysmotility | 4 | 2 | 0.074 |
| constipation | 2 | nd |  |
| dysarthria | 2 | 0 | 1 |
| HTAP | 1 | 0 | 1 |
| **TUMOR OF THE NEURAL CREST** |  |  |  |
| tumor neural crest | 24 | 0 | **0.049** |
| psoas tumor | 0 | 1 | 0.085 |
| **HYPOVENTILATION** |  |  |  |
| acure respiratory failure | 11 | 2 | 0.304 |
| cyanotic episodes | 7 | 0 | 1 |
| central hypoventilation | 28 |  |  |
| obstructive apnea | 15 | 2 | 0.613 |
| **PSYCHATRIC** |  |  |  |
| hallucinations | 3 | 1 | 0.308 |
| major anxiety | 4 | 1 | 0.372 |
| psychosis | 2 | 0 | 1 |
| flat affect | 4 | 0 | 1 |
| mood disorders | 0 | 1 | 0.085 |
| catatonia | 0 | 1 | 0.085 |
| **BEHAVIORAL PROBLEM** |  |  |  |
| tiredness | nd | 2 |  |
| aggressivness | 7 | 1 | 0.539 |
| hyperactivity | 2 |  |  |
| irritability emotional lability | 3 | 1 | 0.308 |
| school difficulties | nd | 2 |  |
| **NEUROLOGICAL** |  |  |  |
| seizures | 3 | 1 | 0.308 |
| narcolepsy | 2 | 0 | 1 |
| hypersomnolence | 6 | 0 | 1 |
| developmental disorder | 9 | 0 | 0.574 |
| ataxia | 0 | 1 | 0.085 |
